# Effect of spironolactone and benazepril on furosemide‐induced diuresis and renin‐angiotensin‐aldosterone system activation in normal dogs

**DOI:** 10.1111/jvim.16097

**Published:** 2021-03-13

**Authors:** Darcy Adin, Clarke Atkins, Gabrielle Wallace, Allison Klein

**Affiliations:** ^1^ College of Veterinary Medicine University of Florida Gainesville Florida USA; ^2^ College of Veterinary Medicine North Carolina State University Raleigh North Carolina USA; ^3^ College of Veterinary Medicine University of Illinois Urbana Illinois USA

**Keywords:** aldosterone antagonist, angiotensin‐converting enzyme inhibitor, continuous rate infusion, diuretic resistance, neurohormone, urine production

## Abstract

**Background:**

Diuretic braking during furosemide continuous rate infusion (FCRI) curtails urine production.

**Hypothesis:**

Renin‐angiotensin‐aldosterone system (RAAS) activation mediates braking, and RAAS inhibition will increase urine production.

**Animals:**

Ten healthy purpose‐bred male dogs.

**Methods:**

Dogs received placebo, benazepril, or benazepril and spironolactone PO for 3 days before a 5‐hour FCRI (0.66 mg/kg/h) in a 3‐way, randomized, blinded, cross‐over design. Body weight (BW), serum creatinine concentration (sCr), serum electrolyte concentrations, PCV, and total protein concentration were measured before PO medications, at hours 0 and 5 of FCRI, and at hour 24. During the FCRI, water intake, urine output, urine creatinine concentration, and urine electrolyte concentrations were measured hourly. Selected RAAS components were measured before and after FCRI. Variables were compared among time points and treatments.

**Results:**

Diuretic braking and urine production were not different among treatments. Loss of BW, hemoconcentration, and decreased serum chloride concentration occurred during FCRI with incomplete recovery at hour 24 for all treatments. Although unchanged during FCRI, sCr increased and serum sodium concentration decreased at hour 24 for all treatments. Plasma aldosterone and angiotensin‐II concentrations increased significantly at hour 5 for all treatments, despite suppressed angiotensin‐converting enzyme activity during benazepril background treatment.

**Conclusions:**

The neurohormonal profile during FCRI supports RAAS mediation of diuretic braking in this model. Background treatment with benazepril with or without spironolactone did not mitigate braking, but was well tolerated. Delayed changes in sCr and serum sodium concentration and incomplete recovery of hydration indicators caused by furosemide hold implications for clinical patients.

**Abbreviations**
ACE: angiotensin‐converting enzyme
AG: anion gap
BP: blood pressure
BUN: blood urea nitrogen
BW: body weight
CHF: congestive heart failure
Cl^−^: chloride
FCRI: furosemide continuous rate infusion
${\mathrm{HCO}}_{3}^{-}$: bicarbonate
K^+^: potassium
Na^+^: sodium
${\mathrm{PO}}_{4}^{-}$: phosphorus
RAAS: renin‐angiotensin aldosterone system
sCr: serum creatinine
sNa : sK: serum sodium to potassium ratio
TS: total solids
UAldo : C: urine aldosterone to creatinine ratio
uNa : uK: urine sodium to potassium ratio

## 1 INTRODUCTION

Furosemide continuous rate infusion (FCRI) has been shown to cause more urine production than bolus administration of the same dose in normal dogs and normal horses^1^, ^2^, ^3^ but, these studies found that urine production decreased after several hours of the infusion (so‐called “diuretic braking”). Renin‐angiotensin‐aldosterone system (RAAS) activation has been proposed as a mechanism to explain diuretic braking in these acute models.^2^, ^4^ Restoration of diuretic responsiveness in this setting may benefit patients with congestive heart failure (CHF) by accelerating resolution of congestive signs, minimizing hospitalization time and cost, and decreasing patient suffering. However, any method that successfully maintains substantial, prolonged urine production has the potential to adversely affect the kidneys and produce undesired effects on serum electrolyte concentrations, associated with the attendant volume depletion.

Because RAAS stimulation may be mechanistically important to the phenomenon of diuretic braking, and because RAAS inhibitors may protect against adverse effects of diuretics, such as electrolyte depletion and other harmful aspects of diuretic‐induced RAAS activation,^5^ these medications are reasonable candidates to modulate diuretic braking. A multimodal approach to RAAS inhibition has been shown to be beneficial to outcome in many studies.^6^, ^7^, ^8^, ^9^, ^10^ Although the long‐term benefit and safety of angiotensin‐converting enzyme (ACE) inhibitors and spironolactone for the treatment of stable CHF has been shown in several studies,^10^, ^11^, ^12^, ^13^ the safety and ability of these RAAS inhibitors to mitigate diuretic braking during FCRI have not been studied. Additionally, although biochemical variables during and immediately after FCRI discontinuation have been evaluated, renal function and serum electrolyte concentrations in the short‐term (recovery) period after diuresis are unknown. We hypothesized that concurrent PO administration of RAAS inhibitors concurrently with FCRI would promote urine production without adverse biochemical effects, thus providing evidence for RAAS activation as a mediator of diuretic braking.

## 2 METHODS

### 2.1 Dogs and protocol summary

The Institutional Animal Care and Use Committee at the North Carolina State University Veterinary Hospital approved this study (protocol #17‐075‐O). The study consisted of a 3‐way crossover design utilizing 10 healthy, male, purpose‐bred dogs, for a total of 30 dog studies. Treatment order was randomized and investigators were blinded to treatment. Each dog underwent all 3 treatments before FCRI, with a 17‐day washout period between crossovers.

**FIGURE 1:**
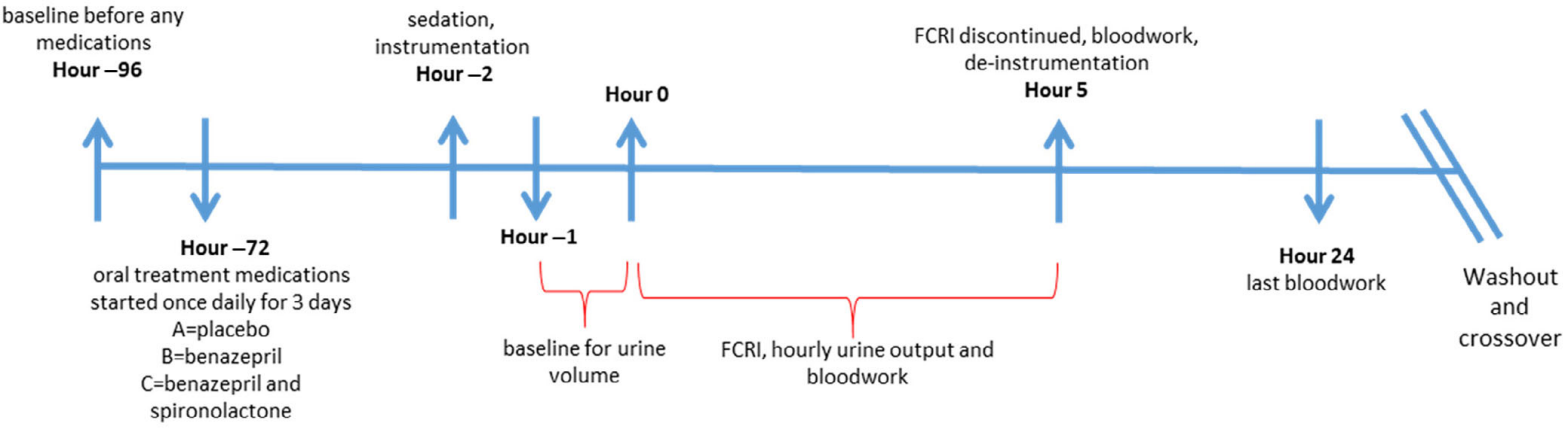
Study timeline. FCRI, furosemide continuous rate infusion

Treatments consisted of PO medications once daily at 7 am for 3 days before and including the morning of FCRI (3.3 mg/kg administered as an IV infusion over 5 hours) diluted to 2.2% with dextrose 5% in water after a 0.66 mg/kg IV loading bolus of furosemide. Medications were placed in an opaque gel capsule and consisted of cellulose as placebo (treatment A), benazepril 0.25 mg/kg minimum dose (treatment B), and benazepril 0.25 mg/kg and spironolactone 2 mg/kg minimum doses (treatment C). Benazepril and spironolactone dosages were selected based on results of studies indicating favorable modulation of the RAAS utilizing these dosages in dogs.^10^, ^14^, ^15^, ^16^, ^17^ Oral treatments were not continued after the FCRI. Dogs were fed a maintenance canine diet with a sodium (Na^+^) content of 112 mg/100 kcal (Certified Canine Diet 5007, LabDiet, St. Louis, Missouri) twice daily (7 am and 4 pm) before and during the study, except that food was withheld on the morning of baseline blood collection and on the morning of the study. The study timeline is shown in Figure 1.

### 2.2 Instrumentation

Dogs were fasted the morning of each study but PO medications were administered. After body weight (BW) was obtained, 2 peripheral IV catheters (18 gauge) were placed for blood sampling, administration of sedative medication for urinary catheter placement (butorphanol 0.3 mg/kg IV), and administration of furosemide during the study period. A urinary catheter (5‐8F Foley; Mila International, Inc, Erlanger, Kentucky) was passed into the urinary bladder, attached to a closed collection system and sutured to the skin. The urinary bladder was emptied before the baseline period. Dogs were allowed to recover from sedation until they could stand (hour −2 to hour −1) and were continuously monitored throughout the study. Water was available at all times. The FCRI ran for 5 hours and urinary and peripheral catheters were removed at the end of each study infusion (at hour 5).

### 2.3 Data collection

Results for baseline variables (blood urea nitrogen [BUN], serum creatinine [sCr], serum Na^+^, serum potassium [K^+^], serum chloride [Cl^−^], serum phosphorus [${\mathrm{PO}}_{4}^{-}$], serum bicarbonate [${\mathrm{HCO}}_{3}^{-}$], anion gap [AG], PCV, total solids [TS], plasma angiotensin II concentration, BW, and blood pressure [BP]) were obtained in the morning (9 am on day −4). Blood for plasma angiotensin II concentration was obtained again at 3 pm to evaluate diurnal variation,^18^ matching the start (am) and end (pm) of the FCRI on the study day. Venipuncture was performed with the dogs in a standing position. Noninvasive BP by oscillometry (Cardell model 9405, Midmark, Tampa, Florida) was performed using a cuff width of approximately 40% of the tail circumference. The average of 3 measurements varying by <10% for systolic, diastolic, and mean BP was recorded.

Data collection on the day of each study began after recovery from sedation and during the 1‐hour baseline period before the 5‐hour FCRI (hour −1 to 0). Baseline study data (at hour 0 before the start of the FCRI) and at the end of the FCRI (hour 5) included BUN, sCr, serum electrolyte concentrations (Na^+^, K^+^, Cl^−^, ${\mathrm{PO}}_{4}^{-}$), ${\mathrm{HCO}}_{3}^{-}$, AG, PCV, TS, BW, BP, hourly urine volume (per kg BW), plasma angiotensin II concentration, urine aldosterone concentration, urine creatinine concentration, urine Na^+^ concentration, and urine K^+^ concentration. Urine volume, plasma aldosterone concentration, urine aldosterone concentration, and urine creatinine concentration were measured hourly during the 5‐hour FCRI. Final data collection was performed 24 hours after the start of the FCRI and included blood collection for BUN, sCr, serum electrolyte concentrations (Na^+^, K^+^, Cl^−^, ${\mathrm{PO}}_{4}^{-}$), ${\mathrm{HCO}}_{3}^{-}$, AG, PCV, TS, BW, and BP. Residual serum samples were used to measure ACE activity by radioenzymatic assay and both chymase activity and ACE activity by equilibrium analysis at hour 0, hour 5, and hour 24. Residual samples for hour −96 were not available for these analyses.

### 2.4 Blood testing

Biochemical testing was performed on site on the same day as sampling. Urine, serum, and plasma samples for remaining tests were frozen at −80°C until analysis, with special handling as described below for plasma angiotensin II samples.

#### 2.4.1 Biochemical tests

Biochemical analysis of serum and urine samples for BUN, sCr, and serum electrolyte concentrations was performed immediately using an automated analyzer (Roche Cobas C501, Indianapolis, Indiana) at the clinical pathology laboratory of the North Carolina State University Veterinary Hospital.

#### 2.4.2 Aldosterone analysis

Aldosterone concentrations (pmol/L) in plasma and urine were measured using a commercially available radioimmunoassay kit (Siemens Medical Diagnostic Solutions, Los Angeles, California), at a veterinary diagnostic laboratory (Diagnostic Center for Population and Animal Health, Michigan State University, Lansing, Michigan) following manufacturer's instructions as previously described.^19^, ^20^ Urine creatinine concentration (mmol/L) was measured using a standard colorimetric assay on the same samples at the same laboratory.

#### 2.4.3 Angiotensin II analysis

A commercially available protease inhibitor was utilized to prevent generation of or degradation of angiotensin II ex vivo in plasma (Angiotensin II Inhibitor Cocktail #9000681, Cayman Chemical, Bertin Pharma, Montigny‐le‐Bretonneux, France) as previously described.^16^ The protease inhibitor was activated by mixing vials A and B and placing the mixture into cold EDTA tubes at a ratio of 30 μL to 1 mL of blood (1 : 33). Blood was added to the EDTA tubes within 30 minutes of activating the protease inhibitor and tubes were immediately centrifuged (20 minutes, 3000*g*, +4°C). Plasma was withdrawn and placed into cold plastic cryo‐tubes and snap frozen using liquid nitrogen within 10 minutes, for immediate storage at −80°C until analysis. A commercially available enzyme‐linked immunoassay was used to assay plasma samples for angiotensin II concentrations in pg/mL (Angiotensin II Enzyme Immunoassay Kit #A05880.96 wells, SPI Bio, Bertin Pharma, Montigny‐le‐Bretonneux, France).

#### 2.4.4 ACE activity by radioenzymatic assay

Activity of ACE (U/L) was directly measured in serum using a radioenzymatic assay (Bühlmann Laboratories AG, Schönenbuch, Switzerland) as previously described (Mayo Clinic, Rochester, Minnesota).^21^, ^22^

#### 2.4.5 Calculations

Body weight change was calculated by comparing BW to that of the previous time point. Absolute BW was not used for group and time comparisons because of the size differences among dogs. Urine aldosterone amount (μg) was indexed to uCr amount (grams) to generate UAldo : C, an indicator of RAAS, under the assumption that renal aldosterone elimination remains constant.^23^, ^24^ Serum sodium to potassium ratio (sNa : sK) and urine sodium and potassium ratio (uNa : uK) were calculated as physiologic indicators of the effect of aldosterone on serum and urine electrolyte concentrations, respectively.^4^, ^25^, ^26^ The percentage change in sCr between hours 0 and 5 and between hours 0 and 24 was calculated. Fractional excretion of sodium (FE Na) was calculated using the following equation: FE Na (%) = 100 × ([urinary Na^+^ × sCr]/[serum Na^+^ × uCr]).

### 2.5 Statistical analysis

Generalized linear mixed effect modeling was performed (SAS software version 9.4, Cary, North Carolina) to evaluate for differences among treatments for repeated hourly and time point measurements. One‐way analysis of variance was used to assess for differences in urine volume among treatments with variables of time, treatment group and time : group interaction after confirming normally distributed residuals and homogeneity of variances. Least square means were estimated for measured variables and data are presented as mean and SD when normally distributed. Tukey's method was utilized to adjust *P* values for multiple comparisons and adjusted *P* values are reported where indicated. Power calculation, based on a previous study,^2^ was performed a priori and indicated that 9 dogs would be necessary to identify a significant difference in urine production among treatments with an alpha of .05 and power of 0.8. Data shown reflect 10 dogs for all variables except ACE activity by radioenzymatic assay (Table 1). Significance was set at *P* < .05.

**TABLE 1:** Median and interquartile ranges for ACE activity (ACE; U/L) measured by radioenzymatic assay at hours 0, 5, and 24 for treatments A, B, and C are shown

	ACE (U/L) hour 0	ACE (U/L) hour 5	ACE (U/L) hour 24	*P* value
Treatment A	22.1 (19.3‐26.9); n = 9	25.0 (20.5‐37.3); n = 7	32.1 (25.7‐36.1); n = 9	.11
Treatment B	2.5 (2.5‐8.9)^^^; n = 10	4.5 (2.5‐10.2); n = 8	25.0 (8.7‐30.2)^*^; n = 9	.002
Treatment C	2.5 (2.5‐7.2)^^^; n = 9	2.5 (2.5‐9.4)^^^; n = 9	18.9 (15.1‐24.0)^*^ ^,^ ^#^ ^,^ ^^^; n = 8	<.001
*P* value	<.001	.005	.008	

*Note:* n = the number of samples available for analysis at each time point. *P* values in the right column indicate comparison among time points. *P* values in the bottom row indicate comparison among treatments. A = placebo before furosemide continuous rate infusion (FCRI), B = benazepril before FCRI, C = benazepril and spironolactone before FCRI. Hour 0 is after 3 days of PO treatment but before FCRI. Hour 5 is at the end of the FCRI. Hour 24 is 24 hours after the start of FCRI.

Abbreviation: ACE, angiotensin‐converting enzyme.

^*^ *P* < .05 compared to hour 0.

^#^ *P* < .05 compared to hour 5.

^^^ *P* < .05 compared to treatment A.

## 3 RESULTS

Study dogs were clinically healthy, intact males, consisting of 8 hound dogs and 2 beagles. Median BW was 23.4 kg (range, 8.7‐29.1 kg) and median age was 44 months (range, 18‐87 months).

### 3.1 Urine volume and natriuresis

**FIGURE 2:**
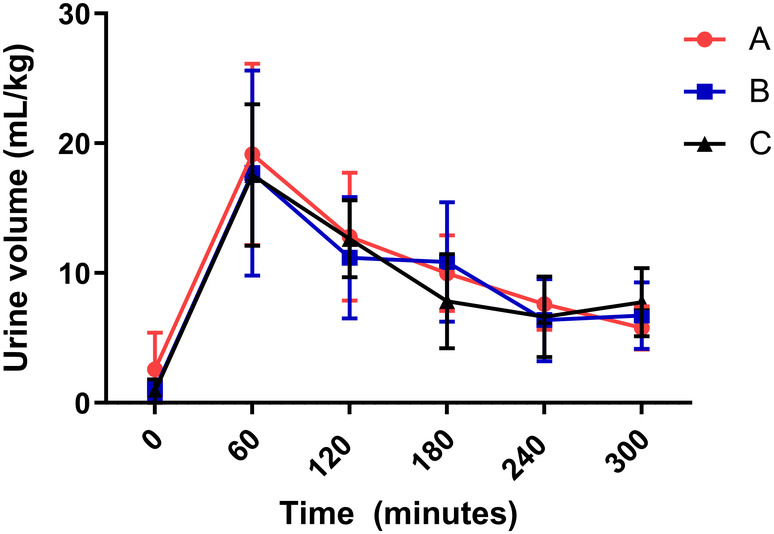
Hourly urine volume (mean ± SD) during furosemide continuous rate infusion (FCRI) for all 3 treatment groups. A = placebo before FCRI, B = benazepril before FCRI, C = benazepril and spironolactone before FCRI. *P* < .001 over time, *P* = .55 among treatments

**FIGURE 3:**
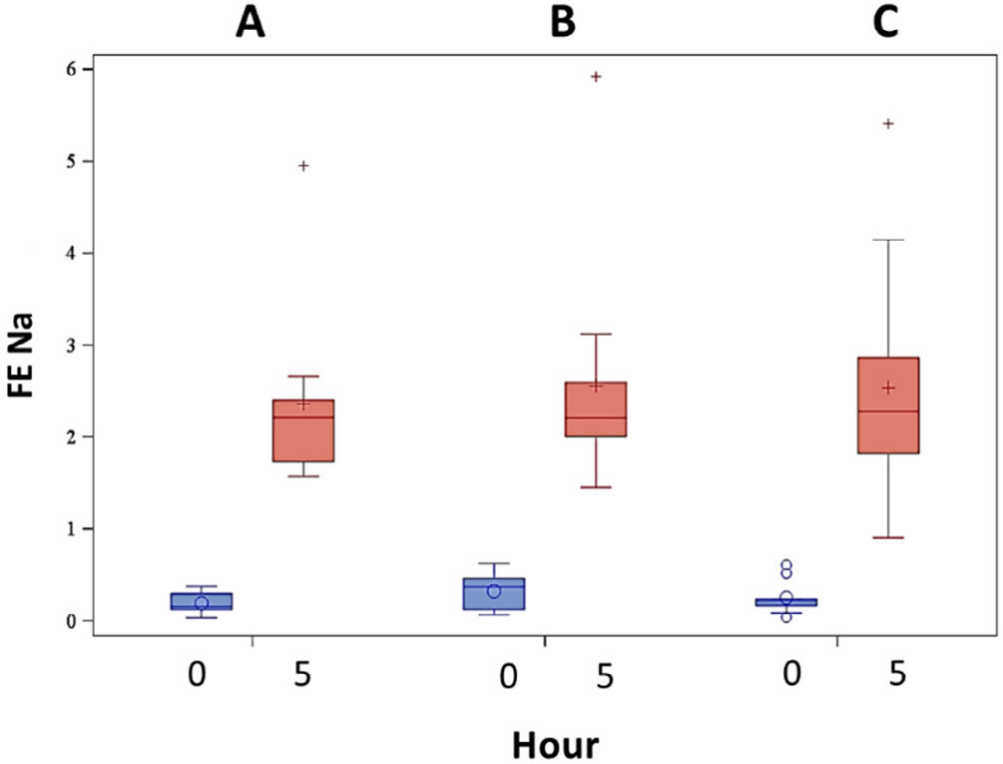
Fractional excretion of sodium before (hour 0) and at the end of (hour 5) furosemide continuous rate infusion (FCRI) is shown for all 3 treatments. A = placebo before FCRI, B = benazepril before FCRI, C = benazepril and spironolactone before FCRI. *P* < .001 over time, *P* = .81 among treatments. For each plot, the colored box represents the 25th to 75th percentile, the bisecting line is the median, the symbol within the box is the mean, and the whiskers are the 5th to 95th percentiles

Hourly and total urine volume (per kg BW) increased over time for all treatments (*P* < .001) in the same pattern as previously noted, with a decrease in urine volume starting at hour 2,^1^, ^2^ but no differences were noted among treatments (*P* = .55 for hourly urine volume, *P* = .59 for total urine volume; Figure 2). The FE Na^+^, used to indicate diuretic resistance when <0.2%, increased from hour 0 to hour 5 for all treatment groups, with no difference among treatment groups (*P* < .001 over time, *P* = .81 among treatments; Figure 3). All hour 5 FE Na^+^ results were >0.2%.^26^, ^27^, ^28^

### 3.2 Clinical variables

**FIGURE 4:**
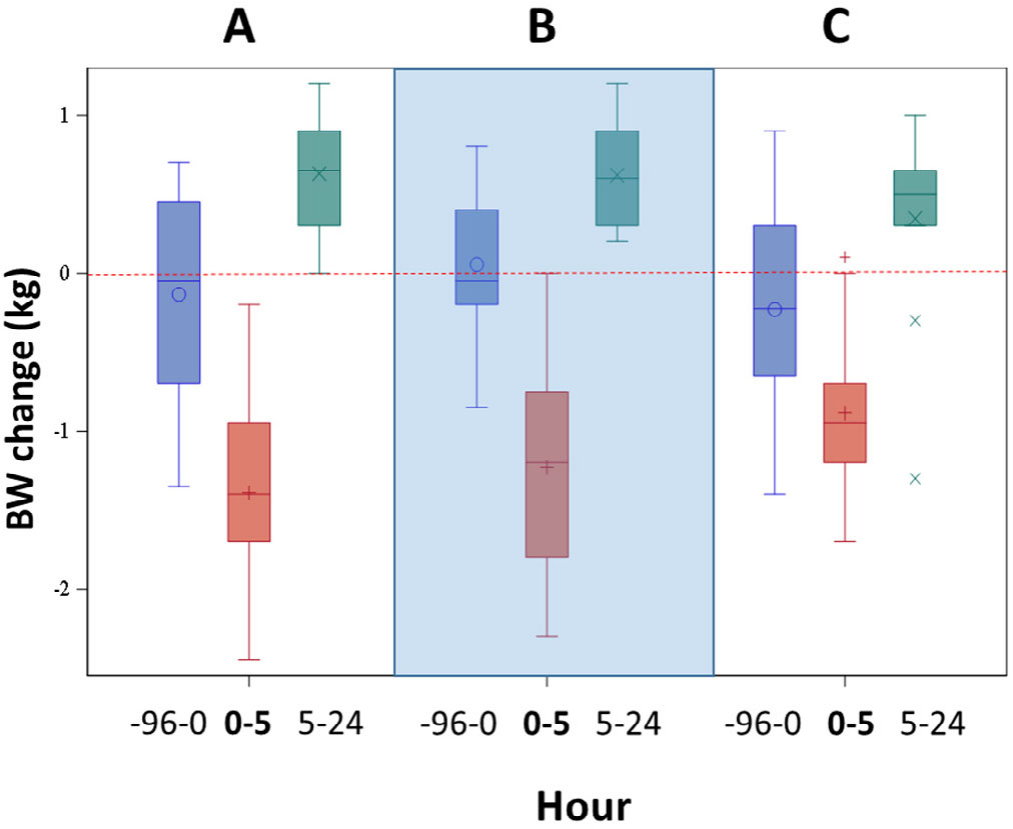
The change in body weight (BW) at each time point compared to the previous time point is shown for each treatment. Data are expressed as the change in BW during each time period (not absolute BW), thus datapoints below the dashed red line indicate weight loss and datapoints above the dashed red line indicate weight gain relative to the previous time point. Blue boxes represent the BW change from hour −96 to hour 0 (PO medications had been administered, but preceding furosemide continuous rate infusion [FCRI]); red boxes represent the BW change from hour 0 to hour 5 (during FCRI); and green boxes represent the BW change between hour 5 and hour 24 (no treatment given). The dashed red line at 0 represents no change. The large shaded box visually separates the treatments. A = placebo before FCRI, B = benazepril before FCRI, C = benazepril and spironolactone before FCRI. *P* < .001 over time, *P* = .6 among treatments. For each plot, the colored box represents the 25th to 75th percentile, the bisecting line is the median, the symbol within the box is the mean, and the whiskers are the 5th to 95th percentiles

Absolute BW and change in BW at each time point were significantly different over time for all treatments (*P* < .001) but were not different among treatments (*P* = 1.0 for BW and *P* = .61 for BW change; Figure 4). Body weight did not return to baseline values by the next day. Blood pressure (systolic, diastolic, mean) did not differ among treatments (*P* > .5 all). Systolic, diastolic, and mean BP were significantly higher at hour −96 (baseline before PO treatments) compared to other hours for all treatments (*P* < .001), but did not change significantly after hour 0 (during the FCRI or at hour 24).

**FIGURE 5:**
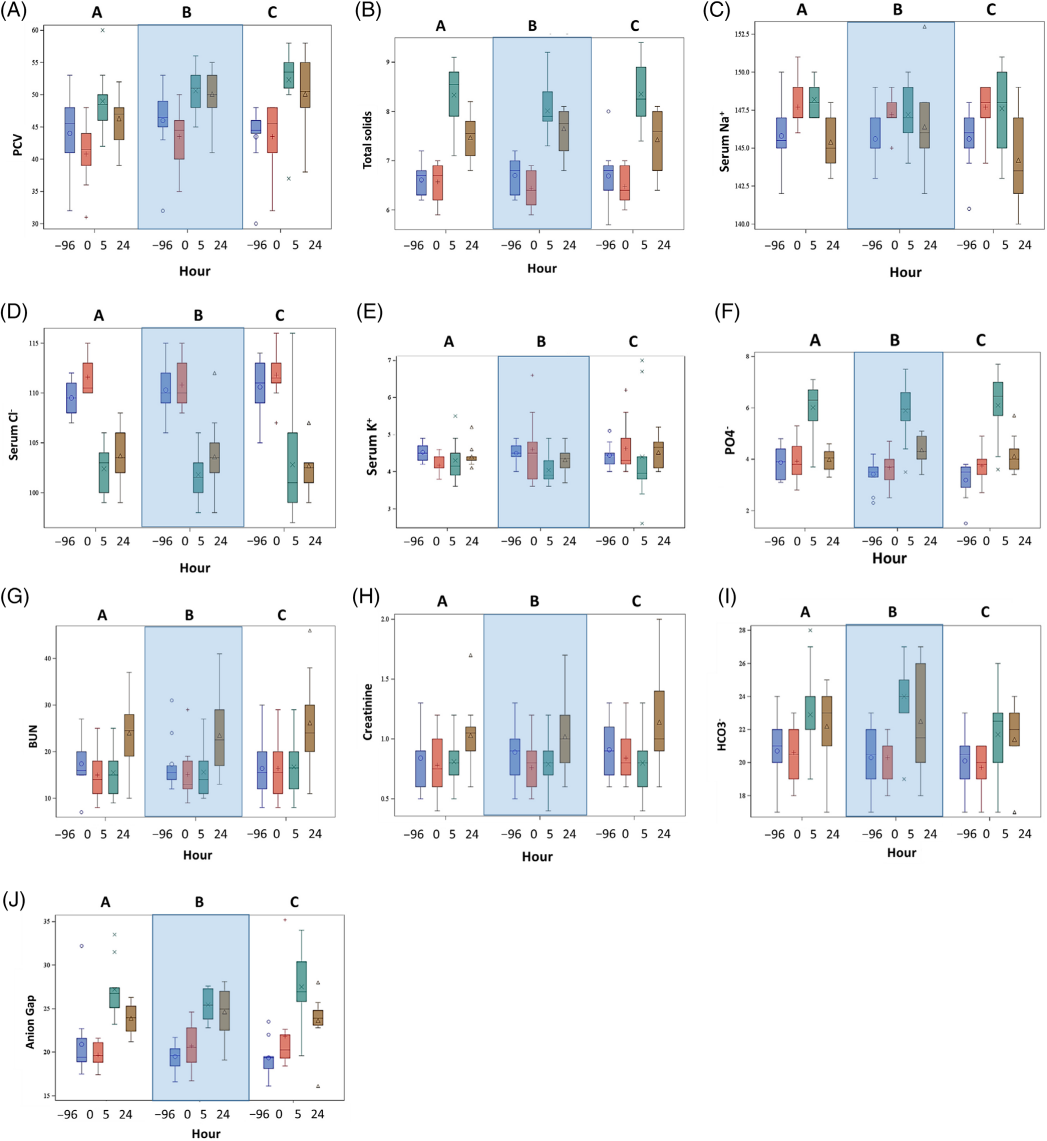
Laboratory variables are shown at each study time point for all 3 treatments. The large shaded box visually separates the treatments. A = placebo before furosemide continuous rate infusion (FCRI), B = benazepril before FCRI, C = benazepril and spironolactone before FCRI. Hour −96 is before PO treatments were started. Hour 0 is after 3 days of PO treatment but before FCRI. Hour 5 is at the end of the FCRI. Hour 24 is 24 hours after FCRI initiation. For each plot, the colored box represents the 25th to 75th percentile, the bisecting line is the median, the symbol within the box is the mean, and the whiskers are the 5th to 95th percentiles. Statistically significant differences among time points were present for all variables regardless of treatment (*P* < .001) except serum potassium concentration (K^+^) for which no differences over time were noted (*P* = .4). Hours that are significantly different from the others are noted below in each graph description with adjusted *P* values. Treatment differences were not noted for any variable (*P* > .3). A, PCV, reference range 35% to 55%. Hours −96 and 0 were both significantly different from hours 5 and 24 (*P* < .001 all). B, Total solids, reference range 6.5 to 8.0 g/dL. Hours −96 and 0 were both significantly different from hours 5 and 24 (*P* < .001 all). Hour 5 was different from hour 24 (*P* < .001). C, Serum sodium (Na^+^) concentrations, reference range 140 to 156 mmol/L. Hours 0 and 5 were significantly different from hour 24 (*P* < .001 both) and from hour −96 (*P* = .005 and.002, respectively). D, Serum chloride (Cl^−^) concentrations, reference range 108 to 122 mmol/L. Hours −96 and 0 were different from hours 5 and 24 (*P* < .001 all). E, Serum K^+^ concentrations, reference range 4.0 to 5.3 mmol/L. No differences between hours were noted. F, Serum phosphorus (${\mathrm{PO}}_{4}^{-}$) concentrations, reference range 2.5 to 5.6 mg/dL. Hours −96, 0, and 24 were different from hour 5 (*P* < .001 all). Hour −96 was different from hour 24 (*P* = .003). G, Blood urea nitrogen (BUN), reference range 6 to 26 mg/dL. Hours −96, 0, and 5 were different from hour 24 (*P* < .001 all). Hour −96 was different from hour 0 (*P* = .03). H, Serum creatinine, reference range 0.7 to 1.5 mg/dL. Hours −96, 0, and 5 were different from hour 24 (*P* < .001 all). Hour 0 and 5 were different from hour −96 (*P* = .005 and .009, respectively). I, Serum bicarbonate (${\mathrm{HCO}}_{3}^{-}$) concentrations, reference range 18 to 26 mmol/L. Hour 0 was different from hours 5 and 24 (*P* < .001 and *P* = .003, respectively). Hour −96 was different from hours 5 and 24 (*P* < .001 and *P* = .01, respectively). J, Anion gap, reference range 11.2 to 19.9. Hours −96 and 0 were different from hours 5 and 24 (*P* < .001 all except *P* < .001 for hour 5 compared to hour 24)

Figure 5 shows the blood variables for each hour for all 3 treatments. Increases in PCV and TS occurred during the FCRI for all treatment groups, with incomplete return to baseline at 24 hours (*P* < .001 over time for both, *P* = .41 among treatments for PCV and *P* = .94 among treatments for TS). A significant decrease in serum Na^+^ concentration occurred at hour 24 for all treatments (*P* < .001 over time, *P* = .66 among treatments). A significant decrease in serum Cl^−^ concentration occurred at hour 5 for all treatment groups and did not return to baseline at hour 24 (*P* < .001 over time, *P* = .94 among groups). A significant increase in serum ${\mathrm{PO}}_{4}^{-}$ concentration was noted at hour 5 for all treatments (*P* < .001 over time, *P* = .79 among treatments). Similarly, a significant increase in serum ${\mathrm{HCO}}_{3}^{-}$ concentration occurred at hour 5 for all treatments (*P* < .001 over time, *P* = .26 among treatments) and in AG at hour 5 for all treatments (*P* < .001 over time, *P* = .78 among treatments). No change in serum K^+^ concentration over time (*P* = .4) or among treatments (*P* = .58) was found.

Both BUN and sCr increased significantly (*P* < .001 over time), evident only at hour 24 for all treatments (Figure 5). However, no difference was found among groups (*P* > .8 for both). The percentage change in sCr from hour 0 to 5 and from hour 0 to 24 was not different among treatments (*P* = .2 and *P* = .9, respectively). The mean (SD) percentage increase in sCr from 0 hour to 24 hour was 23.9% (12.4) for all 3 treatments.

Despite statistically significant changes in all blood variables (except serum K^+^ concentration) over time, mean results remained within the reference ranges for all variables except serum Cl^−^ concentration, TS, and AG. Biochemical results for all treatments reflected movement toward hypochloremic metabolic alkalosis, with a resulting AG increase at hour 5, and persisting at hour 24.

### 3.3 Neurohormonal analyses

**FIGURE 6:**
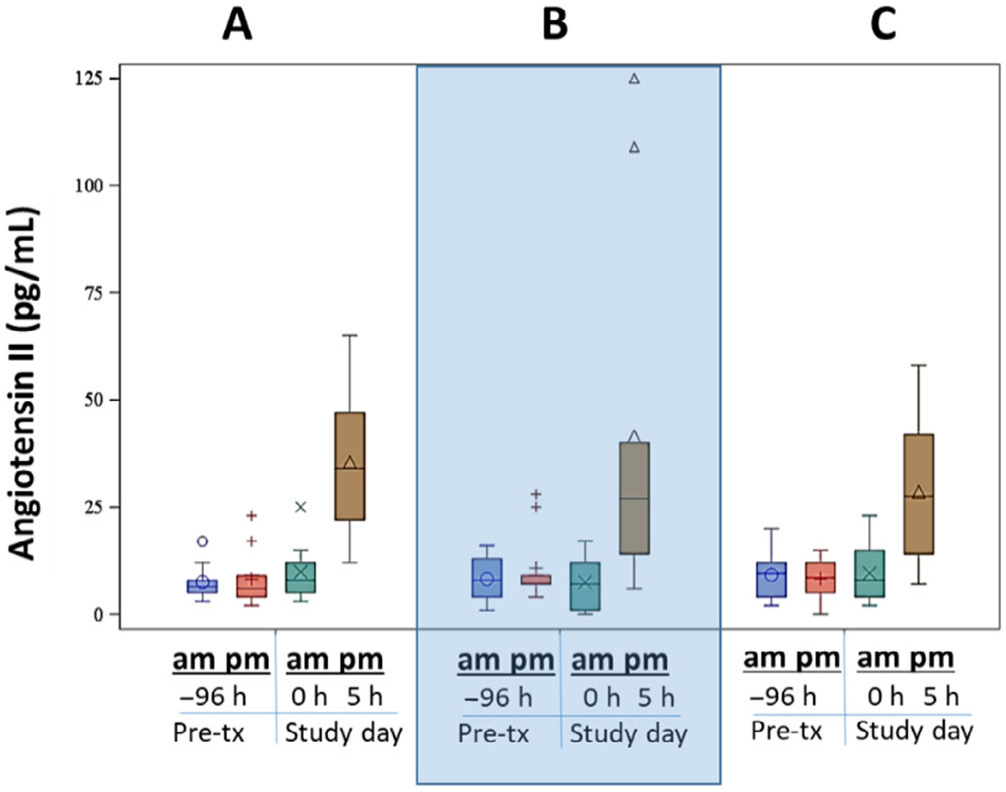
Plasma angiotensin II concentrations at baseline (morning and afternoon before any study treatments) and at hour 0 and 5 (before and after furosemide continuous rate infusion [FCRI]). The large shaded box visually separates the treatments. A = placebo before FCRI, B = benazepril before FCRI, C = benazepril and spironolactone before FCRI. For each plot, the colored box represents the 25th to 75th percentile, the bisecting line is the median, the symbol within the box is the mean, and the whiskers are the 5th to 95th percentiles. No within day or between day differences were noted (*P* = 1.0 both). No differences among treatment groups (*P* = .82) were noted. The angiotensin II concentration significantly increased from hour 0 to hour 5 for all treatments (*P* < .001)

Plasma angiotensin II concentrations did not show diurnal variation (*P* = 1.0 between morning and afternoon at hour −96) or day‐to‐day variation (*P* = 1.0) but were significantly increased at the end of the FCRI (hour 5) compared to before the FCRI (hour 0; *P* < .001) for all treatments (*P* = .82 among treatments; Figure 6).

**FIGURE 7:**
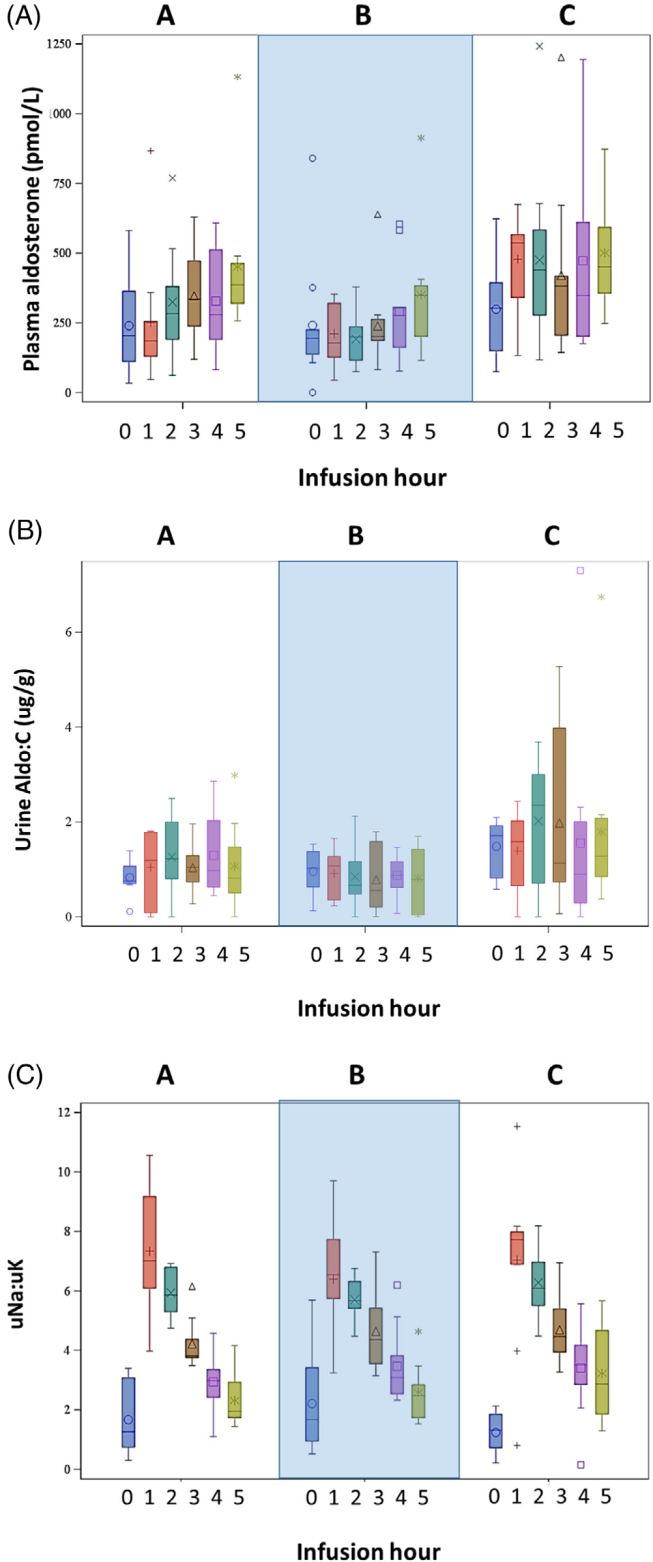
Hourly aldosterone‐related variables are shown during the furosemide continuous rate infusion (FCRI). A = placebo before FCRI, B = benazepril before FCRI, C = benazepril and spironolactone before FCRI. The large shaded box visually separates the treatments. For each plot, the colored box represents the 25th to 75th percentile, the bisecting line is the median, the symbol within the box is the mean, and the whiskers are the 5th to 95th percentiles. A, Plasma aldosterone increased over time for all treatments (*P* < .001). Hour 5 was significantly different from hour 0 (*P* < .001) and hour 1 (*P* = .04). No difference among treatments was noted (*P* = .08). B, Urine aldosterone : creatinine did not change over time (*P* = .72) or among treatments (*P* = .08). C, The uNa : uK ratio was significantly different over time (*P* < .001) with a pattern similar to urine production; however, no treatment differences were present (*P* = .74). All hours were significantly different from each other (*P* < .05) except hour 0 and hour 5 (*P* = .06) and hour 4 and hour 5 (*P* = .42)

Hourly plasma aldosterone concentrations increased over time for all treatments (*P* < .001; Figure 7). Differences in plasma aldosterone concentrations did not reach statistical significance among treatments (*P* = .08 for treatment effect, *P* = .09 for interaction between treatment and time). Whereas UAldo : C increased in dogs receiving treatment C, the difference among treatments did not reach statistical significance (*P* = .08) and no overall time effect was observed (*P* = .72). The uNa : uK changed over time (*P* < .001) in concert with diuresis, but no treatment effect was noted (*P* = .74). The uNa : uK was >1.0 at all time points during diuresis, indicative of diuretic responsiveness.^25^, ^28^ The sNa : sK increased over time (*P* < .008, with hour 5 significantly higher than hours −96 and 24), but no treatment effect was identified (*P* = .92).

Direct assessment of ACE activity by radioenzymatic assay at hours 0, 5, and 24 for dogs with residual serum available is shown in Table 1. Sufficient sample volumes were available for 7 to 10 dogs at each hour of each treatment. Activity of ACE at hour 0 (after 3 days of PO pretreatment but before FCRI) was significantly lower for treatments involving benazepril (B and C) compared to placebo (A; *P* < .001). After FCRI, treatment C (benazepril and spironolactone) ACE activity remained significantly lower than treatment A (placebo) ACE activity (*P* = .02 at hour 5 and *P* = .04 at hour 24). The ACE activity at hour 5 for treatment B (benazepril) was not significantly different when compared to treatment A (placebo; *P* = .06), and no significant increase compared to its own baseline was observed at hour 0 (*P* = .6). No difference in ACE activity was identified among time points for treatment A (placebo; *P* = .11).

## 4 DISCUSSION

Our results support RAAS activation as a mechanism of diuretic braking in this FCRI model using healthy dogs, despite finding that background PO administration of RAAS suppressive medications did not mitigate diuretic braking. The increase in plasma angiotensin II concentrations at the end of the furosemide infusion and the increase in hourly plasma aldosterone concentrations throughout the infusion support the involvement of these neurohormones in curtailing diuresis even with continued IV diuretic administration and previously demonstrated furosemide presence in the urine.^4^ Plasma angiotensin II concentration was measured only at hours 0 and 5, and thus its exact relationship to hourly plasma aldosterone concentrations and urine production was not elucidated in our study. Previous studies in our laboratory have shown that braking occurs despite continued furosemide presence in the urine.^2^, ^4^ In the current study, diuretic braking occurred despite significant suppression of ACE activity for treatments B and C, both of which included benazepril. It is unknown whether a higher benazepril dosage or frequency of administration would have suppressed ACE activity more or would have affected urine production. The lack of differences in angiotensin II and aldosterone concentrations among the treatment groups raises the possibility that PO RAAS suppression using ACE inhibitors may be inadequate to overcome the intense RAAS stimulation that this FCRI model produces.^29^ The RAAS cascade is complex, with multiple enzymatic pathways for formation and degradation of metabolites other than ACE.^5^, ^30^, ^31^ The information from our study sheds light on a potential mechanism of angiotensin II and aldosterone breakthrough in the specific setting of intense RAAS stimulation caused by FCRI, which may be able to overcome or circumvent the effects of RAAS suppressive medications.

Although true baseline ACE activity (before PO treatments) was not available to allow calculation of the percentage of ACE suppression in each treatment group, the treatments for which benazepril was administered before FCRI (treatments B and C) showed only 10% of the ACE activity after 3 days of medication compared to treatment A (no benazepril). The low ACE activity with benazepril administration in our study is consistent with other studies that found significant ACE suppression with an ACE inhibitor.^15^, ^32^ The reason that ACE activity remained lower for a longer period of time with treatment C (benazepril and spironolactone) is not known, but could be a consequence of the attenuating effect of spironolactone on the catalytic activity of ACE, which has been shown in vitro.^33^ This finding, considered with the protective effect of spironolactone against the maladaptive effects of aldosterone influence, supports the beneficial effect of the combination of an ACE inhibitor and mineralocorticoid receptor antagonist in situations in which the RAAS is stimulated. Our study results suggest that angiotensin II concentrations rapidly increase in response to FCRI‐induced diuresis, potentially resulting from non‐ACE pathway angiotensin II formation by enzymes such as chymase, kallikrein, elastase‐2, cathepsin G, or cathepsin D, or the interplay of angiotensin II generation, degradation, and feedback within complex RAAS pathways.^30^, ^31^ The role and importance of circulating non‐ACE enzymes to the RAAS cascade in dogs is incompletely explored.^34^, ^35^ We consider it reasonable that the increase in aldosterone could be a result of angiotensin II stimulation of the angiotensin type 1 receptor, but nonangiotensin pathways for aldosterone production were not explored in our study.^36^ The increase in aldosterone concentration noted in dogs receiving treatment C likely was influenced by mineralocorticoid receptor blockade.

Ours is the first study in dogs evaluating biochemical variables 24 hours after cessation of IV furosemide administration. Some furosemide‐induced biochemical changes were not evident until the day after the infusion, and some biochemical changes that occurred during the infusion had not returned to baseline at 24 hours. Concurrent PO administration of RAAS inhibitors did not significantly affect these changes, which were found for all treatments (A, B, and C). This observation indicates that the major driver for these findings likely was the loop diuretic, and not RAAS‐inhibiting medications. Because no differences were noted among treatments, the biochemical results of our study support the safety of RAAS‐suppressive medications administered before FCRI in healthy dogs, but the implications for CHF management in dogs have not been elucidated. Although changes were relatively mild in this model, the discovery of serum biochemical abnormalities indicating volume depletion and electrolyte derangements appearing the day after the FCRI raise concern about the potential for similar changes in clinical patients. Dogs hospitalized for treatment of acute CHF may have pre‐existing renal dysfunction or may receive higher doses or longer infusions of furosemide to manage their condition and therefore may be at higher risk for azotemia and electrolyte abnormalities. The average sCr increase in these dogs at hour 24 approached the International Renal Interest Society definition of acute kidney injury (http://www.iris-kidney.com/guidelines/grading.html). Higher doses, longer infusions, or even high‐dose PO furosemide may predispose to similar or more marked changes in sCr, particularly in older patients. Although the increases in sCr in the dogs of our study likely were based in hemodynamics (decreased glomerular filtration as a result of diuretic‐induced volume depletion),^37^ acute kidney injury can encompass both functional and structural insults (http://www.iris-kidney.com/guidelines/grading.html). Repeated subclinical episodes of acute kidney injury could contribute to cardiovascular‐renal disorders in dogs, which can contribute to poor outcome or death.^38^, ^39^, ^40^ To our knowledge, this 24‐hour time point for evaluating adverse effects of diuretics has not been studied previously in dogs or people receiving IV furosemide aside from a small study that found delayed hyponatremia in people receiving IV furosemide for transurethral prostate resection.^41^

The model used in our study of normal dogs has limited applicability to dogs with naturally occurring CHF, but nevertheless provides mechanistic information to drive future clinical studies. The neurohormonal RAAS and sympathetic nervous system profiles of dogs with CHF are activated at baseline, which was not the case for these healthy dogs. Accordingly, the different fluid balance and neurohormonal state between healthy dogs and dogs with CHF could influence the response to RAAS suppression and diuresis with FCRI. Equilibrium analysis of the entire RAAS cascade would have been helpful in understanding the source of increased angiotensin II at hour 5, but we were unfamiliar with this methodology during study planning. Moreover, equilibrium analysis utilizing liquid chromatography and mass spectroscopy may have provided more accurate aldosterone concentrations in light of specificity concerns for radioimmunoassay testing. Additional time points after FCRI discontinuation would have provided more information regarding RAAS involvement in the unexpected findings of prolonged biochemical changes and hydration recovery. Despite these limitations, the results of our study provide insight into the response of the RAAS, renal function, and electrolyte balance during and after intense RAAS stimulation caused by FCRI, with and without RAAS‐suppressive treatment with relatively low‐dose ACE inhibition and mineralocorticoid antagonism.

## 5 CONCLUSIONS

Oral administration of RAAS inhibitors did not prevent the diuretic braking that occurred with FCRI in this healthy dog model, but the observed curtailment in urine production appeared to be at least partially mediated by some components of the RAAS. The biochemical changes that occurred in response to FCRI are attributable only to the furosemide and therefore administration of RAAS inhibitors before FCRI appears safe in healthy dogs. Results of other chronic studies support the use of RAAS inhibitors, especially mineralocorticoid receptor antagonists, to protect against the maladaptive effects of aldosterone when the RAAS is activated; our results support the safety of this approach in the setting of acute CHF treatment. The delayed biochemical effects secondary to IV furosemide observed in our study warrant evaluation in dogs with naturally occurring CHF to determine if similar effects occur with IV diuresis in a clinical setting.

## CONFLICT OF INTEREST DECLARATION

Dr Adin is a consultant for Ceva Santé Animale and Boehringer Ingelheim. She has received speaking honoraria and travel reimbursements from Ceva Santé Animale. Dr Atkins has received funding from and has consulted for Ceva Santé Animale, Boehringer Ingelheim, and Vetoquinol. Dr Wallace and Ms Klein do not have conflicts of interest to report.

## OFF‐LABEL ANTIMICROBIAL DECLARATION

Authors declare no off‐label use of antimicrobials.

## INSTITUTIONAL ANIMAL CARE AND USE COMMITTEE (IACUC) OR OTHER APPROVAL DECLARATION

Approved by North Carolina State University IACUC, protocol #17‐075‐O.

## HUMAN ETHICS APPROVAL DECLARATION

Authors declare human ethics approval was not needed for this study.

## References

[1] Adin DB , Taylor AW , Hill RC , Scott KC , Martin FG . Intermittent bolus injection versus continuous infusion of furosemide in normal adult greyhound dogs. J Vet Intern Med. 2003;17:632‐636. 14529128 10.1111/j.1939-1676.2003.tb02493.x

[2] Adin D , Atkins C , Papich M , et al. Furosemide continuous rate infusion diluted with 5% dextrose in water or hypertonic saline in normal adult dogs: a pilot study. J Vet Cardiol. 2016;19:44‐56. 27840084 10.1016/j.jvc.2016.09.004

[3] Johansson AM , Gardner SY , Levine JF , et al. Furosemide continuous rate infusion in the horse: evaluation of enhanced efficacy and reduced side effects. J Vet Intern Med. 2003;17:887‐895. 14658727 10.1111/j.1939-1676.2003.tb02529.x

[4] Adin D , Atkins C , Papich MG . Pharmacodynamic assessment of diuretic efficacy and braking in a furosemide continuous infusion model. J Vet Cardiol. 2018;20:92‐101. 29483040 10.1016/j.jvc.2018.01.003

[5] Ames MK , Atkins CE , Pitt B . The renin‐angiotensin‐aldosterone system and its suppression. J Vet Intern Med. 2019;33:363‐382. 30806496 10.1111/jvim.15454 PMC6430926

[6] Pitt B , Pfeffer MA , Assmann SF , et al. Spironolactone for heart failure with preserved ejection fraction. N Engl J Med. 2014;370:1383‐1392. 24716680 10.1056/NEJMoa1313731

[7] Zannad F , McMurray J , Krum H , et al. Eplerenone in patients with systolic heart failure and mild symptoms. N Engl J Med. 2011;364:11‐21. 21073363 10.1056/NEJMoa1009492

[8] Pitt B , Zannad F , Remme WJ , et al. The effect of spironolactone on morbidity and mortality in patients with severe heart failure. N Engl J Med. 1999;341:709‐717. 10471456 10.1056/NEJM199909023411001

[9] Borgarelli M , Ferasin L , Lamb K , et al. DELay of Appearance of sYmptoms of canine degenerative mitral valve disease treated with spironolactone and benazepril: the DELAY study. J Vet Cardiol. 2020;27:34‐53. 10.1016/j.jvc.2019.12.002. 32032923

[10] Bernay F , Bland J , Haggstrom J , et al. Efficacy of spironolactone on survival in dogs with naturally occurring mitral regurgitation caused by myxomatous mitral valve disease. J Vet Intern Med. 2010;24:331‐341. 20102506 10.1111/j.1939-1676.2009.0467.x

[11] BENCH (BENazepril in Canine Heart disease) Study Group . The effect of benazepril on survival times and clinical signs of dogs with congestive heart failure: results of a multicenter, prospective, randomized, double‐blinded, placebo‐controlled, long‐term clinical trial. J Vet Cardiol. 1999;1:7‐18. 10.1016/S1760-2734(06)70025-X 19081317

[12] The COVE Study Group . Controlled clinical evaluation of enalapril in dogs with heart failure: results of the Cooperative Veterinary Enalapril Study Group. J Vet Intern Med. 1995;9:243‐252. 8523321 10.1111/j.1939-1676.1995.tb01075.x

[13] Lefebvre HP , Ollivier E , Atkins CE , et al. Safety of spironolactone in dogs with chronic heart failure because of degenerative valvular disease: a population‐based, longitudinal study. J Vet Intern Med. 2013;27:1083‐1091. 23869534 10.1111/jvim.12141

[14] King J , Mauron C , Kaiser G . Pharmacokinetics of the active metabolite of benazepril, benazeprilat, and inhibition of plasma angiotensin‐converting enzyme activity after single and repeated administrations to dogs. Am J Vet Res. 1995;56:1620‐1628. 8599524

[15] King JN , Christinaz C , Strehlau G , Hornfeld J . Effect of benazepril and pimobendan on serum angiotensin‐converting enzyme activity in dogs. J Vet Pharmacol Ther. 2018;41:485‐489. 29392741 10.1111/jvp.12475

[16] Mochel JP , Fink M , Peyrou M , Soubret A , Giraudel JM , Danhof M . Pharmacokinetic/pharmacodynamic modeling of renin‐angiotensin aldosterone biomarkers following angiotensin‐converting enzyme (ACE) inhibition therapy with benazepril in dogs. Pharm Res. 2015;32:1931‐1946. 25446774 10.1007/s11095-014-1587-9

[17] Guyonnet J , Elliott J , Kaltsatos V . A preclinical pharmacokinetic and pharmacodynamic approach to determine a dose of spironolactone for treatment of congestive heart failure in dog. J Vet Pharmacol Ther. 2010;33:260‐267. 20557443 10.1111/j.1365-2885.2009.01130.x

[18] Mochel JP , Fink M , Bon C , et al. Influence of feeding schedules on the chronobiology of renin activity, urinary electrolytes and blood pressure in dogs. Chronobiol Int. 2014;31:715‐730. 24654920 10.3109/07420528.2014.897711

[19] Lantis AC , Ames MK , Werre S , Atkins CE . The effect of enalapril on furosemide‐activated renin – angiotensin – aldosterone system in healthy dogs. J Vet Pharmacol Ther. 2015;38:513‐517. 25771846 10.1111/jvp.12216

[20] Ames MK , Atkins CE , Eriksson A , Hess AM . Aldosterone breakthrough in dogs with naturally occurring myxomatous mitral valve disease. J Vet Cardiol. 2017;19:218‐227. 28576479 10.1016/j.jvc.2017.03.001

[21] Meurs KM , Olsen LH , Reimann MJ , et al. Angiotensin‐converting enzyme activity in Cavalier King Charles Spaniels with an ACE gene polymorphism and myxomatous mitral valve disease. Pharmacogenet Genomics. 2018;28(2):37‐40. 29200408 10.1097/FPC.0000000000000322

[22] Meurs KM , Stern JA , Atkins CE , et al. Angiotensin‐converting enzyme activity and inhibition in dogs with cardiac disease and an angiotensin‐converting enzyme polymorphism. J Renin Angiotensin Aldosterone Syst. 2017;18:147032031773718. 10.1177/1470320317737184 PMC5843865 29069972

[23] Ames MK , Atkins CE , Lantis AC , et al. Evaluation of subacute change in RAAS activity (as indicated by urinary aldosterone:creatinine, after pharmacologic provocation) and the response to ACE inhibition. J Renin Angiotensin Aldosterone Syst. 2016;17:1‐12. 10.1177/1470320316633897 PMC5843907 27009288

[24] Atkins CE , Lantis AC , Ames MK , Gardner SY . Utility of urinary aldosterone measurement in quantitating RAAS activation. J Vet Pharmacol Ther. 2012;35:512‐515. 22966900 10.1111/jvp.12011

[25] Eudy RJ , Sahasrabudhe V , Sweeney K , et al. The use of plasma aldosterone and urinary sodium to potassium ratio as translatable quantitative biomarkers of mineralocorticoid receptor antagonism. J Transl Med. 2011;9:1‐11. 10.1186/1479-5876-9-180 PMC3305907 22017794

[26] Doering A , Jenkins CA , Storrow AB , et al. Markers of diuretic resistance in emergency department patients with acute heart failure. Int J Emerg Med. 2017;10(1):17. 28484958 10.1186/s12245-017-0143-x PMC5422212

[27] Verbrugge FH . Editor's Choice‐Diuretic resistance in acute heart failure. Eur Hear J Acute Cardiovasc Care. 2018;7:379‐389. 10.1177/2048872618768488 29897275

[28] Adin D , Kurtz K , Atkins C , Papich MG , Vaden S . Role of electrolyte concentrations and renin‐angiotensin‐aldosterone activation in the staging of canine heart disease. J Vet Intern Med. 2020;34:53‐64. 31769114 10.1111/jvim.15662 PMC6979094

[29] Harada K , Ukai Y , Kanakubo K , et al. Comparison of the diuretic effect of furosemide by different methods of administration in healthy dogs. J Vet Emerg Crit Care. 2015;25:364‐371. 10.1111/vec.12301 25885944

[30] Uehara Y , Miura S , Yahiro E , et al. Non‐ACE pathway‐induced angiotensin II production. Curr Pharm Des. 2013;19:3054‐3059. 23176219 10.2174/1381612811319170012

[31] Danser AHJ . Local renin–angiotensin systems: the unanswered questions. Int J Biochem Cell Biol. 2003;35:759‐768. 12676161 10.1016/s1357-2725(02)00178-4

[32] Toutain PL , Lefebvre HP , King JN . Benazeprilat disposition and effect in dogs revisited with a pharmacokinetic/pharmacodynamic modeling approach. J Pharmacol Exp Ther. 2000;292:1087‐1093. 10688627

[33] Stoll D , Yokota R , Sanches Aragão D , Casarini DE . Both aldosterone and spironolactone can modulate the intracellular ACE/ANG II/AT1 and ACE2/ANG (1‐7)/MAS receptor axes in human mesangial cells. Physiol Rep. 2019;7:e14105. 31165585 10.14814/phy2.14105 PMC6548847

[34] Fujii Y , Orito K , Muto M , Wakao Y . Modulation of the tissue renin‐angiotensin‐aldosterone system in dogs with chronic mild regurgitation through the mitral valve. Am J Vet Res. 2007;68:1045‐1050. 17916008 10.2460/ajvr.68.10.1045

[35] Yamane T , Fujii Y , Orito K , Osamura K , Kanai T , Wakao Y . Comparison of the effects of candesartan cilexetil and enalapril maleate on right ventricular myocardial remodeling in dogs with experimentally induced pulmonary stenosis. Am J Vet Res. 2008;69:1574‐1579. 19046003 10.2460/ajvr.69.12.1574

[36] Lefebvre H , Duparc C , Naccache A , et al. Paracrine regulation of aldosterone secretion in physiological and pathophysiological conditions. Vitam Horm. 2019;109:303‐339. 30678861 10.1016/bs.vh.2018.10.001

[37] Tabaru H , Finco DR , Brown SA , Cooper T . Influence of hydration state on renal function of dogs. Am J Vet Res. 1993;54:1758‐1764. 8250404

[38] Martinelli E , Locatelli C , Bassis S , et al. Preliminary investigation of cardiovascular‐renal disorders in dogs with chronic mitral valve disease. J Vet Intern Med. 2016;30:1612‐1618. 10.1111/jvim.14524. 27717188 PMC5032878

[39] Pouchelon JL , Atkins CE , Bussadori C , et al. Cardiovascular‐renal axis disorders in the domestic dog and cat: a veterinary consensus statement. J Small Anim Pract. 2015;56:537‐552. 26331869 10.1111/jsap.12387 PMC4584495

[40] Uechi M , Matsuoka M , Kuwajima E , et al. The effects of the loop diuretics furosemide and torasemide on diuresis in dogs and cats. J Vet Med Sci. 2003;65:1057‐1061. 14600341 10.1292/jvms.65.1057

[41] Donatucci CE , Deshon GE , Wade CE , et al. Furosemide‐induced disturbances of renal function in patients undergoing TURP. Urology. 1990;35:295‐300. 2181769 10.1016/0090-4295(90)80148-g

